# Understanding mixed emotions in organized helping through emotionography

**DOI:** 10.3389/fpsyg.2023.1236148

**Published:** 2023-10-12

**Authors:** Alexa Hepburn, Jonathan Potter

**Affiliations:** School of Communication and Information, Rutgers, The State University of New Jersey, New Brunswick, NJ, United States

**Keywords:** emotionography, mixed emotion, conversation analysis, discursive psychology, crying, upset, laughter

## Abstract

**Introduction:**

Emotionography studies emotion: (a) as it occurs naturally in display, reception, attribution, and avowal; (b) within and across diverse stretches of interaction and varied institutional contexts; (c) grounded purposefully in the perspectives of the interactants as those perspectives are displayed in real-time through unfolding talk; (d) using materials that are recorded and transcribed in sufficient precision to capture the granularity consequential for the interactants. We overview contemporary research on “mixed emotion” highlighting theoretical and methodological issues and explore the potential of emotionography as a generative alternative.

**Methods:**

The analysis will use contemporary conversation analysis and discursive psychology to illuminate the workings of organized helping using a collection of recordings from a child protection helpline all of which include laughter alongside crying.

**Results:**

Analysis shows, on the one hand, how crying and upset display the caller's stance on the trouble being reported, and mark its action-relevant severity; on the other, how laughter manages ongoing parallel issues such as advice resistance. We show that the “mixture” is public and pragmatic, displaying different concerns and stances, and dealing with different issues; all is in the service of action.

**Discussion:**

When analyzing the specifics of interaction, the concept of “mixed emotion” loses clarity, and it is more accurate to observe competing pragmatic endeavors being pursued in an intricately coordinated fashion. These practices would not be captured by conventional emotion measurement tools such as scales, vignettes, or retrospective interviews. Broader implications for theories of emotion and methods of emotion research are discussed.

## Introduction

Research on emotion in psychology and the social sciences overwhelmingly relies on scales, inventories, vignettes, experimental simulations, reconstructions from field notes, and, occasionally, qualitative interviews; sometimes, these traditional measuring methods are correlated with physiological or neuroscientific measures. There remains a significant absence of research that explores the development of emotional episodes in their natural, real-time settings—places where emotions are exhibited, received, acknowledged, and ascribed. Although there is an abundance of studies and numerous theories surrounding emotion, a precise observational science for “emotion in the wild” is largely missing. For a simple illustration, if one were to examine the *Handbook of Emotions* (Barrett et al., 2016) for a recent summary of emotion research, one would find no examples of concrete, documented displays of emotion, attributions, or avowals connected to different theoretical perspectives.

There is, therefore, a need for and space for an emotionography.^1^ This has the following characteristics:

1. a comprehensive study of emotions as they occur naturally, encompassing their display, reception, attribution, and avowal;
2. within and across various types of interactions and social contexts;
3. grounded resolutely in the perspectives of the interactants as their perspectives are displayed in real-time through unfolding talk; and
4. using recorded and transcribed materials in sufficient detail and accuracy to capture the granularity consequential for the interactants.

Such an approach will address how emotions are intertwined with actions in interaction, sometimes performing independent actions, sometimes modulating them, and sometimes obstructing them. Emotionography offers an observational science for emotion that explores what occurs during actual concrete emotional displays and episodes and their reconstruction through interlocutors' descriptions and versions as parts of further actions. It is not meant to replace current work on emotion but to foster a new perspective and catalyze a different dialogue.

The roots of this perspective come from conversation analysis and discursive psychology, which are disciplinary areas that provide many of the theoretical and analytic resources we are drawing on (Edwards, 1997, 1999; Hepburn, 2004; Potter and Hepburn, 2010; Peräkylä and Sorjonen, 2012; Weatherall and Robles, 2021). We have not attempted to summarize these perspectives here (see Hepburn and Potter, 2021, on conversation analysis and Wiggins, 2016, on discursive psychology). Rather, we have illustrated what is involved in adopting such an approach to emotion with examples from organized helping and, at the same time, demonstrated the benefit of taking a systematic approach to emotion itself, an emotionography. To illustrate the value and power of this approach, we considered a recent discussion of “mixed emotion” from a range of more mainstream perspectives. We have demonstrated how our action- and interaction-focused analysis offers an alternative account for at least some situations of mixed emotions and their relevance to organized helping.

### Mixed emotion

Let us begin by considering current understandings and presuppositions about mixed emotions. When characterizing mixed emotions with opposite valences, such as “happy” and “sad,” some researchers have discussed oscillations between different states (e.g., Russell and Carroll, 1999), while others have argued for the inseparability of positive and negative emotions (e.g., Cacioppo and Berntson, 1994). In discussions of attitudes, such as liking or disliking, emotional states with a particular valence are generally assumed to be more enduring (e.g., Cacioppo et al., 1997). The prevailing assumption is that there is a duality between the expression of mixed or blended emotions (e.g., Scherer, 1998) and their psychological substrate, and the aim of researchers should, therefore, be to access it in as pure a form as possible. This has led to the development of research instruments, such as questionnaires, which use bipolar scales ranging from positive to negative emotions (e.g., Russell and Carroll, 1999); examples include studies based on the observation of simultaneous smiling and frowning facial expressions (e.g., Griffin and Sayette, 2008) and studies employing Ekman and Friesen's (1978) “facial action coding system.”

In their critique of other methods employed to access the “pristine inner experience” of mixed emotions, such as questionnaires, Hurlburt and Heavey (2015) observed that such methods are influenced more by presuppositions and judgments than the experiences themselves. Consequently, they produce statistical data that generate misleading results. Heavey et al. (2017) contend that the quality of the data used to inform theories of emotion should be a primary concern. To access “pristine inner experiences” of mixed emotions and rectify this issue, Heavey et al. (2017) employed a method referred to as “descriptive experience sampling” (DES). This method entails providing participants with a beeper that goes off at random intervals during their daily lives. After each stimulus, participants are asked to make brief notes on their thoughts or feelings. This is followed by an interview, usually within 24 h, in which investigators collaborate with participants to elaborate on their experiences at each moment and produce “high-fidelity descriptions.” They conclude that approximately 1–5% of moments contain either “blended feelings,” where feelings of opposite valences, such as sadness and happiness, appear to constitute a single feeling, or “mixed emotions,” in which a positive and negative feeling “exist separately” but are felt simultaneously. One of their examples is a participant's description of feeling happy about leaving work but annoyed about having to return the next day.

As the authors acknowledge, there are many problems with methods aiming to surmise emotions from self-descriptions. The DES attempts to overcome some of these by having participants work with actual events that happened to them (as opposed to vignettes or descriptions on a questionnaire). However, problems arise in conceiving alternative ways of doing systematic and rigorous observational work when it comes to emotion, largely because researchers are operating with the basic assumption that feelings are accessible states of mind that can be accurately intuited and described *post-hoc*. However, the examples given contain constructions of emotion and experience in relation to events that are generated via interviews, and interaction-focused researchers have highlighted various problems and limitations associated with data generated in this way (e.g., Edwards, 1997; Potter and Hepburn, 2012; Silverman, 2017).

Psychologists and evolutionary theorists, despite the importance in their disciplines of close observation, have few tools with which to systematically explore social interaction. Therefore, research in these areas has been done without the benefit of a close interactional analysis of the phenomena. When we utilize emotionography, grounded in conversation analysis and discursive psychology, we can observe that the language of the psychological sciences has insufficient purchase on emotion episodes in practice and can easily provide circular explanations for interactional phenomena, for example, evidence for the biological/neurological basis for emotional expression is that people have been observed to produce emotional facial expressions at the same time. A straightforward link has been made between the facial expression and the emotion because the researcher has no apparatus for looking closely at their interactional location.

### Interactional research on emotion

Working with recorded interactions, studies from the perspectives of DP and conversation analysis (CA) have focused on the role of “emotion displays” (Hepburn, 2004; Ruusuvuori, 2012), “affective stances” (e.g., Couper-Kuhlen, 2009), and “action modulation” (Shaw et al., 2013) in achieving specific interactional goals. This then forms a basis for developing new ways of understanding emotion as constitutive of action formation or, to paraphrase Weatherall and Robles (2021), how emotions are made to do things. Such studies have demonstrated the importance of various features, such as word order, gaze, gestures, facial cues, silence, breathiness, and prosodic delivery (including pitch, volume, and emphasis), in shaping both the conveying and interpretation of emotion displays. These features are combined with lexical choices and turn-taking patterns in sequential relation to one another. Crucially, these underpin the performance of action and interaction. While this level of detail is beyond the capability of research participants to precisely recall and reconstruct, it is crucial to understand the role of emotions in speakers' everyday lives. It starts to provide a basis for exploring emotion and action in contexts such as psychotherapy and other forms of organized helping (e.g., Muntigl, 2020).

From a discursive psychological (DP) perspective, the underlying assumptions of emotion research in the social sciences and the methods used to generate data are problematic. For instance, Edwards (1997, 1999, 2005) has demonstrated how emotional avowals and attributions are constructed in and for discursive practices and used as resources for offering justifications, making complaints, and assigning blame. Thus, it is important to understand their role in social interaction rather than assuming that descriptions of emotions on scales or in interviews are simply neutral representations of inner life. Potter and Hepburn (2005, 2012) have similarly highlighted the challenges associated with interview-based studies, including the failure of researchers to comprehend the interactional work that participants engage in when constructing descriptions of their experiences. This study presents the view that emotions, whether mixed or not, are phenomena that are constructed in and for interaction and, on occasion, interfere with or modulate ongoing action. Interaction is a principal, perhaps even the principal space, where emotions are live and consequential. It will also build on existing conversational analytic research on emotion, specifically in relation to laughing and crying.

### Interactional research on laughing

Jefferson's (1984) study of laughter during troubles was one of the earliest to demonstrate that laughter should not be viewed solely as an indicator of happiness or amusement. Instead, Jefferson revealed how trouble tellers, for example, use laughter to present themselves as trouble-resistant. Building on this finding, Potter and Hepburn (2010) showed that laughter particles can be interpolated into speech to manage descriptive trouble, for example, the inadequacy of a word when a speaker complains about a child's inappropriate punishment and inserts a laughter particle into the word “punishment,” to both use the word and flag up its problematic status. They also demonstrated how laughter could modify the nature or strength of action, such as when a caller describes a child using the charged term “porker,” while discussing a troubling family living nearby and uses interpolated laughter to soften the problematic nature of the description and display an understanding of its potentially inappropriate use. Building on this, Shaw et al. (2013) showed how post-completion laughter, or laughter at the end of a turn, can modulate its disaffiliative or misaligned features and signal appropriate next actions to recipients. They also emphasized the importance of capturing the specific quality of laughter being used, such as whether it is minimal, quiet, and breathy or louder and longer with exaggerated pitch changes.

### Interactional research on crying

Inspired by Jefferson's study on laughter, Hepburn (2004) initiated a project focused on analyzing episodes of upset in interaction. She advocated for the importance of a detailed transcription of crying and its responses. Meticulous transcription can help us view crying as a collection of loosely related and occasionally escalating practices, much like laughter, and make it open to more specific interactional analysis. As a result, the intricate interactional nature of crying starts to reveal itself. Her work showed that crying can inflect talk, sometimes hinder, intensify, or emphasize it and occasionally replace it rather than appear as an action or a set of actions on its own. This makes the uptake of crying particularly challenging, as it requires orienting toward something that is displayed or the way it is delivered rather than an action, claim, or proposition. Moreover, crying in adults, especially in institutional settings, can give the impression that the crier does not want their state to be part of public discourse, resulting in challenging issues in responding.

Across several projects, (Hepburn and Potter, 2007, 2010, 2021) analyses have explored crying and responses, developing a procedural explication of sympathy and empathy. They observed that sympathetic turns are often delivered with specific prosodic features, such as quieter volume, stretched duration, rising and falling pitch contours, and/or creaky and breathy delivery. In contrast, empathic turns often suspend the routine course of conversation to focus more explicitly on the crying person's distress and may involve formulating their emotional state using phrases such as “this must be frustrating/difficult for you” while downplaying their own possibly problematic entitlement to that understanding with epistemically focused constructions such as “I guess” or using tags (“isn't it”).

This research on crying and laughing as interactional activities serves as a foundation for studying mixed emotions, specifically through the analysis of instances where laughing and crying co-occur in calls to a child protection helpline. Consistent with our emotionography, our focus is not on upset and happiness as internal psychological states but on how laughing and crying work in interaction. We are interested in examining what is observable and publicly available, as it constitutes the lived experience of the participants in our data. We aim to show how this “mixture of emotion” can be understood because of the intertwining of different action projects within the interaction. For our study, examining mixed emotions requires our analysis to be true to the integrity of the interaction we are studying, which requires us to recognize the granularity that is live for the participants and the intricate turn and sequence organization of the unfolding interaction that the interactants orient to. Notably, our goal is to explain precisely what happens in individual cases. This precision is crucial as a prerequisite for any potential generalizations. We believe that substantial work is needed to establish a robust foundation from which generalizations can be derived. We will work on those cases.

### Data

The article discusses examples from a collection of more than 150 calls to a UK child protection helpline. The helpline provides free counseling, information, and advice to anyone concerned about a child at risk of abuse and is staffed by trained social workers. Where they judge it to be warranted, they will make a referral to social services and, in extreme cases, to the police. The current study drew on a subset of 15 calls, collected between September 2000 and June 2003, that involved audible upset. This corpus served as the basis for initial research on crying and the application of these findings (for the latter, see Hepburn and Potter, 2004; Hepburn et al., 2014). Of this corpus, five calls contained laughter or laughter particles in or alongside the crying. All participants gave full consent for the calls to be recorded and used for various research and teaching purposes, and ethical consent was granted by Nottingham Trent University's ethics committee, following the helpline's own internal consent procedures. All references to names, places, and other identifying features were anonymized.

### Transcription system

The near-universal standard transcription system for interaction research was developed by Jefferson (2004). A full introduction can be found in Hepburn and Bolden (2017), including extensions of the system to focus on crying and upset found in Hepburn (2004). A brief summary of elements of the system that are important for emotion displays can be found in Appendix 1 below. We include some annotations of the examples we use as we go along to help novice readers.

## Analysis

### “Mixed emotion” in a mundane conversation

Before we consider our helpline data, let us start with an example of an interaction from an everyday US English language phone call between friends. This can introduce some of the issues to be explored and indicate the point of precise transcription. Penny has called Pat, having heard that her house has burned down—a situation that is likely to have some emotional traction. Pat gives Penny a detailed account of how the disaster unfolded. The extract below starts 3 min and 6 sec into an 11-min call. You may find it useful to refer to the summary of transcription conventions to assist in following our discussion. Even in this short extract, there is much to interest emotion researchers. Our focus, however, will be on the role of laughter in Penny's declaration that she is crying, as found in lines 9–11.

**1. Houseburning 3.06-3.24(from Heritage, 2011, p. 175)**

01 PAT:   =It happened within minutes. .hh Within a half hour the
02               house wz go:ne I guess,=
03 PEN:   =Oh:hh go:d,
04 PAT:   So it's jist l[i:ke, we wouldn', we just would'na been=
05 PEN:                 [.hhh
06 PAT:   =here. hh yihkno:w,
07 PEN:   [  O   h   h   h   ] b a: b y.]
08 PAT:   [There's no way ih wz] ih wz jus]:, we're jist lucky I guess:,
**09 PEN: .****hhhh** Okay waid[amidnit  I   ]don'know if your **cry(h)in** b't

**10 PAT:               [(hhh y'know.)]**

**11 PEN: £****I****=hhh(h)ahhhm£ uh** **hu****:h .hhh=**
12 PAT:   =.hh I wz guh- I- middle a'the night la-ast night I
13               wannhhhidhhhtihh c(h)all (h)y(h)ou .mhhh! I [ said ] oh: I=
14 PEN:                                                                                                             [uh hh-]
15 PAT:   =wish I wz at lunch so I c'go talk tuh Penn(h)y hh[hh .hhh
16 PEN:                                         [Yehh(h)ehh

One characteristic of both laughter and upset is that both involve a high degree of aspiration or “breathiness.” We can see this in the multiple inhalations (.hhh), e.g., line 9, and exhalations (hhh). This breathiness is also interpolated into the delivery of individual words and phrases; e.g., “I am” on line 11 is rendered as “£I=hhh(h)ahhhm£” on line 11. However, there are further features of delivery that allow more disambiguation. There is a form of delivery that conversation analysts call “smile voice” because it sounds as if the speaker is smiling while talking, something recognizable even in a phone call. Smile voice is marked by enclosing such delivery between £ symbols. Thus, “£I=hhh(h)ahhhm£” is delivered with a smile voice. Furthermore, we can see the post-completion laugh particles immediately following: uh h**u:h**.

As Shaw et al. (2013) showed, post-position laughter immediately following action can be used to disarm or modulate an action that might be hearable as disaffiliated or challenging without completely retracting it. Here, Penny is invoking the idea that she might be more upset than Pat, whose house just burned down, so by adding post-completion laughter, she conveys the extent to which her friend's problem affects her while neutralizing some of the problematic features of her claim. It is interesting to note the laughter particle on the word “crying” in line 9. Potter and Hepburn (2010) suggested that these interpolated laughter particles function to mark some limitation or problem with the specific words within which they are interpolated. Again, the problem relates to Penny's claim to be crying when Pat has the primary right to own the upset (Sacks, 1992; Heritage, 2011). Although Penny is hearably breathy, and her pronunciation shows signs of minor nasal blockage (e.g., “waid[ami**d**nit” on line 9), there are no other hearable features of crying, such as sobbing or tremulous delivery.

In this example, we observe self-attributions of being upset, which are reminiscent of those found in more traditional studies on emotional distress (a discussion of self-report questions used in crying inventories can be found in Hepburn, 2004). We want to emphasize the intricate nature of emotion as a dynamic phenomenon within live interactions. In many ways, this complexity falls through the net cast by traditional methods, raising the question of whether the simplified outcomes they often yield are more a product of the methodology than an accurate reflection of real-life emotional experiences.

For instance, if Penny were asked to rate her level of empathy on a scale (e.g., Hogan, 1969; Hojat et al., 2001), she might choose the highest rating of “strongly agree” to indicate how easily she can put herself in others' shoes. If she were asked to complete the PANAS-X scale, would she select both “jovial” and “sad” as descriptors in this particular moment? We can see how these self-reported measures tend to oversimplify the complexity inherent in interactional dynamics. If the natural habitat of emotions is within the realm of social interaction, it becomes crucial to unpack the multifaceted emotional intricacies that often go unnoticed in these self-reported and similar research methodologies.

This is precisely what we aim to initiate with a collection of examples highlighting “mixed emotions” from a child protection helpline. By examining these instances, we seek to delve into the richness of emotional experiences that may not be adequately captured in existing studies and methodologies.

### “Mixed emotion” in child protection helpline calls

Child protection helplines are an environment where heightened emotion is common. A caller may be upset about something they have witnessed, say, or a family member may be angry about the treatment of a child by a stepparent. Callers may also be angry and upset about the helpline's inability to be more proactive or to instruct local social services to act for them. When we started working with the helpline in the late 1990s, the call takers nominated crying and upset as the first thing to usefully study (see Hepburn, 2004; Hepburn and Potter, 2007, 2012). For the most part, our studies highlighted the finely tuned skills that the call takers deployed in managing crying and upset (Hepburn et al., 2014). Our focus here is on situations that are candidates for mixed emotions.

Let us start with a relatively straightforward example. The caller (CAL) is calling about her friend who is self-harming. In the process of describing the cuts on her friend's arm, she has, in vernacular terms, become upset, which has led her to take time out of talking. We join the call as the call taker (CT) advises her to encourage her friend to contact a school counselor. Throughout our analysis, various features hearable as elements of crying and upset (U>) or laughter particles (L>) are shown in bold and and arrowed to disambiguate them on the transcript.

**2. JX Self-harming friend 040402 2.54**

01 CT:          The school [may be ab]le to put ‘er in touch
02 CAL: **U>**                            [ **.shihh**  ]
03 CT:          with the school ↑counse↓llor or someone like
04                   that.=a specially [trAIN- ]
05 CAL:                     [Well sh]e has- e- (0.2) she
06            **L>** ↑is actually (.) em a peer **couns(hh)ellhuh**.
07                 (.)
08 CT:       [ She's a peer coun-   ]
09 CAL: **U>** [She **~****deal****s with these~**] **~things which~ sh-**
10             **L>** **uhuh .hhhh**
11                (.)
12 CAL:  Y e ah.
13                (0.2)
14 CT:       Well ↑she needs to have some help herself.=
15                =does[n't s]he:.
16 CAL:       [Yeah.]

The call taker builds her advice project in lines 1–4. In overlap, the caller gives a wet sniff (line 2), which is most likely a legacy from the earlier bout of upset, and then, as it becomes clear that advice is focused on seeking a school counselor, the caller pushes back. She breaks into the call-taker's continued advice delivery on line 5 with some laughter-infused resistance, noting that her self-harming friend is *herself* a peer counselor. The caller delivers the specific word “counselor” with interpolated laughter particles (***cou*ns**(**hh**)**ellhuh**. As we noted earlier, such interpolated laugh particles can mark a limitation or problem with the words they are infused with Potter and Hepburn (2010). The trouble here is with the “counselor” and her friend's existing counselor status. The caller's continuation on line 9 has post-completion laughter on line 10, that is, after the action being delivered is completed. In resisting CT's advice, the caller is building a challenging action, which the post-completion laughter works to disarm. In addition, the particles throughout the specific word “counselor” mark the complexity of her having a role that she should be turning to for help.

Immediately prior to her post-completion laughter on line 10, the caller's continuation of her resistance on line 9 is accompanied by a tremulous delivery, displaying her continued upset. Again, we can see laughter and crying mixed up together in this brief sequence. However, in action terms, they are working in different ways. The upset displays the caller's stance on the trouble she is reporting and the action-relevant severity of that trouble. The laughter is modulating and managing the advice resistance. The “mixture,” then, is public, pragmatic, and in the service of action.

We will develop this further in a more complex example. In the following, the caller is phoning about her 14-year-old daughter, who has been physically and verbally aggressive with her; she proposes, somewhat obliquely, that the daughter be taken into care by social services. The call taker (CT), as is standard in such cases, proposes alternatives, including family therapy, and advises the mother to put more direct time and resources into supporting her daughter. The caller has been resisting this line of advice for the last few minutes and has shown upset at various points during the call. We join the call as the call taker advises the caller to take some time off work to deal with the situation.

**3. NS WO Problem daughter II 100102 10.20**

01 CT:            R:ight.=Would it not be possible for you to maybe
02                     take some lea:ve while-while she's livin [wiv you.]
03 CAL: **U>**                                                                                                    [.**shhn**    ]=
04                     =>W'l l've only< jus' #started this job.=[I mean ]=
05 CT:                                                                                                           [Ri:ght.]=
06 CAL: **U>**   =#uh possible #bu:t y'know it'd be **~unpai:d** ‘n
07 CAL: **L>**         I'm [just **st](h)artin a new mor(hh)tghage,=han=**
09 CT:                    [ Mm:. ]
10 CAL:    =[I .hhh ye know i]ts: (1.3)
11 CT:            =[Ri:ght. Ri:ght. ]
12 CT:            Yeah:.=.HH I mean- ye know at the end of the day i-it's
13                      about priorities isn' it.=an [ye know obvi]ously she:'s
14 CAL:                                 [ I know:. ]
15 CT:            got to come fir:st in all of this.=[because she's (the-)]
16 CAL:                                      [Yeah but if I've got]
17 CAL: **L>**    nowhere to **li(hh)ve** then she sh- .hhh [ye know,]
18 CT:                                                                                                    [ NO::. ]=But=
19 CAL:    =[.hhh ]
20 CT:            =[ye kn]ow I mean social services would be sayin to ↓you:,
21                    (.) ye know, i-u- that (.) th-the job would have to come
22                    secondary.=I mean ultimately [  as I said  ]
23 CAL: **U>**                                                                       [But it **~ca:n']t**.
24                   (.)
25 CAL: **L> >Hh-hhsh-hh<** [In a ] wa:y, i-it c[an't] because I need (.) to:
26 CT:                                        [We:ll]            [Mm. ]
27 CAL: **L>** (.) earn money te **#livehh** .**hhh** ye know,=[An'] she's not willing
28 CT:                              [Mm.]
29 CAL: **L>** she doesn't <want to live> with **m(h)e**[:.=Thi]s is the [thing]
30 CT:                        [ Mm:. ]        [Mm:. ]
31 CAL:    she <doesn't want me.=She hates me.=[She do]esn't want>
32 CT:                        [ Mm. ]
33 CAL: **L> m(hh)e**.=.hhh[hh ]
34 CT:            [But] all of [that needs to be sorted ] out doesn'=
35 CAL: **U>**               **[ An** **I** **~can't** **ma****ke #her.~]**
36 CT:      =i[:t.=I mean it- ]
37 CAL: **U>     [~I** **c****an't ↑make ] [her.~]**

As we have emphasized in the previous two cases, appreciating the actions that are unfolding and the way they are emotionally inflected will involve considerable attention to the specifics of delivery. In line 1, CT reiterates a prior line of advice suggesting that the mother should spend more time working on her relationship with her daughter. This time, she suggests taking time off work as one way to achieve that. The caller resists this advice by claiming to have just started a new job (line 4), meaning that she would not be paid. We can note some subtle elements of possible upset here: First, a short but wet-sounding sniff “.shhn” (line 3); second, the “creaky voice” present in “#started” (line 4) and “#uh” and “#but” (line 6), which can accompany upset but is not a defining element; finally, “~unpaid” (line 6) has the tremulous delivery characteristic of talking through upset. Prior to this, the caller has shown some signs of upset at various points in her initial problem presentation, but not throughout much of CT's subsequent advice-implicative questions [e.g., asking about other family members who could step in and help; see Butler et al. (2010) on the role of advice-implicative interrogatives]. Note that the caller's subtle displays of upset and perhaps vulnerability here are in response to CT's initiating question on lines 1–2, which is designed with a negative interrogative at the beginning. Heritage (2002) has shown that turn initial negative interrogatives can package questions that challenge the assumptions or assertions of recipients by embodying questioners' own contrasting assumptions. By responding in a manner that flags up her own difficulties and helplessness, the mother in this call further pushes back against the unwelcome advice.

Back to extract 3. Another important element of the caller's advice resistance is her laughter-infused “st]ahrtin a new mo(h)r(h):gage' (line 7), which is what the caller offers as a further account for not being able to take leave from her job to look after her daughter. Here, the caller's emphasis on her new mortgage can be heard as embodying a problematic priority: being concerned with material matters more than her daughter's distressing problems: The mother is “starting a new mortgage” at a time when she should instead be prioritizing her daughter. The laughter that infuses this account functions to modulate its action; she is providing an account while flagging attentiveness to its problematic and limited status. By interpolating laughter particles, the mother can keep her problematic descriptions in play.

Potter and Hepburn (2010) also noted how these kinds of laughter-infused turns can manage the following appropriate action, i.e., they have a role not just in managing the descriptive work of a turn but in modulating the action of a turn to shape the subsequent sequence. In the face of the caller's laughter modulating her focus on her mortgage, CT reissues her advice in an idiomatic form in a way that we found characteristic of managing advice resistance on the helpline (Hepburn and Potter, 2011). In this case, “at the end of the day” (line 12) and “it's about priorities isn' it” (line 13), CT also rushes on to spell out what the caller's priorities need to be here: her daughter must come first (line 15).

The caller fights for the conversational floor through the call taker's advice delivery in lines 12–15. The key element in her pushback—that she would have nowhere to live if she followed CT's advice—is again inflected with a laugh particle modulating her challenging resistance, marking the limitations of the extreme formulation “nowhere to live,” and perhaps marking attentiveness to the selfish focus. A similar pattern emerges from line 23. The call taker persists in advice delivery (the caller should not prioritize her job over her daughter), and the caller pushes back, in overlap, with “But it can't.” This has tremulous delivery through the principal word “~ca:n't” and follows this directly with post-completion laughter, which again modulates the challenge and the way it has interrupted CT, and maybe also flagging consciousness of being selfish (Shaw et al., 2013).

From here on, the caller's pushback against CT's advice on lines 25–37 is repeated increasingly emotively and is grounded in the child's perspective—“she doesn't want me.” While the laughter particles through “me” on 29 and 33 similarly modulate her action of strong resistance, she is also describing the breakdown of her relationship with her daughter. Describing the relational trouble of a daughter who does not want to live with her is when more crying elements come in on 35 and 37.

Let us highlight the implications we wish to draw from this analysis. There is considerable conflict in this interaction; in effect, each party has a competing project: the caller hopes that her difficult daughter can be put into the care of social services; the call taker is proposing a re-focus on the daughter's welfare with its necessary sacrifices. Despite the conflict here, there is an intricate interactional choreography, with each party paying close attention to the other's actions. Our focus has been on the role of upset (displayed through creaky voice, elevated pitch and aspiration, and tremulous voice) and laughter particles, both interpolated in words and positioned after turns. What do we make of this? Is this a mixed emotion? If the caller was part of the “descriptive experience sampling” study and her buzzer went off during this sequence, would she say that she was both upset and happy? We cannot know. However, for us, the question is how these two emotional displays might work interactionally. Put simply, the upset underlines the difficult situation of the caller, her need for help, and her difficulty coping in support of the project of social services taking care of her daughter. This upset is not made hearable continually during the interaction but is placed in keywords and in relation to relevant actions on the part of the call taker. The laughter is also interpolated into keywords and follows key turns, showing attentiveness to possible inappropriate selfishness and modulating turns challenging the call taker's advice. The key observation for us is that what might seem like a mysterious emotional mixture of upset and happiness is understandable as the delicate prosecution of unfolding actions.

Let us consider a final helpline example. The following call highlights the fine line between identifying what might definitively be termed laughter particles and managing some kind of descriptive trouble on lines 6–7 and aspiration associated with the upset, which becomes more apparent a few seconds later. The caller here is phoning in to say that she has just found out that her brother-in-law has been sexually abusing children and that others in the family did not tell her at the time, meaning that she inadvertently exposed her children to potential harm.

**4. BN Old abuse 141100 2.55**

01 CT:    Have you just [ re]cently found out about thi:s,
02 CAL:            [so-]
03 CAL:    Er:: e-e- appairently it came up a-a-a-a month ago:,
04      (0.3)
05 CAL:    .hhh Er:m (0.5) an-an to be honest is I find it very
06      strange because appairently .hhh I'm to:ld,=**bhy** **mhhy**
**07**        **dhaughter** that my husband kne:w, (0.2) .hh (0.3)
08        er: some ti:me ago.
09        (0.6)
10 CAL:    And that I was never ↓to:ld,=an I- I'm afraid **I dh-**
**11**        **I ~↑fhind mysehlf very** **an****:grhy.~=~as well as very-~**
**12**        **.hh ↑uhhh ~****v****ery up#s****e****:t.~**
13        (0.3)
14 CAL:    Be ↑**ca**use (0.8) ~a:[hh.hhh]
15 CT:            [it was] kept from you.
16 CAL:    A-w- >↑In some wa:ys,=and what ↑rhisk were my
17      ↑children hha- put at.=

By inflecting the source of her shocking information with breathy laugh particles—being told “bhhy mhhy dhaughter” (lines 6–7)—the caller shows that she understands that it is problematic to be told by her daughter rather than her husband, who knew for so long without telling her. In line 11, there is aspiration in “fhind mysehlf” and “angrhy,” yet it is combined with tremulous delivery and an elevated pitch characteristic of upset. Notably, these delivery features are combined with explicit emotional avowals: “I find myself very angry” and “very upset”; in this way, the caller makes her stance on the news very clear. Her “anger” displays an appropriate moral condemnation of the withholding of information; her “upset” displays her status as a victim of the withholding.

In a discussion of the differences between crying and laughing, Hepburn (2004) noted that, although sobbing may look similar in appearance to laughter in a transcript and may indeed sound similar when isolated as sound files, the participants, as practical analysts of one another's talk, typically do not appear to have trouble distinguishing the two. This can be shown by contrasting the uptake between laughing and crying. For example, laughter may solicit reciprocal laughter (Jefferson, 1979; Glenn, 1989), or when employed to “make light” of a trouble-telling situation, it may be ignored to focus on the serious pursuit of the topic (Jefferson, 1979, 1984). Crying recipients in institutional encounters, on the other hand, may delay their turns, allowing the crying party time to compose themselves, often overtly marking the delay *as* a delay with some version of “take your time,” and, in more extreme cases, adding turns that display sympathy, reassurance, and empathy (Hepburn, 2004; Hepburn and Potter, 2007, 2012).

## Discussion and conclusions

In the field of emotion research, prevailing assumptions have led researchers to seek methods that provide access to the psychological substrate of emotion, treating it as an entity that exists and drives behavior. However, the reliance on research instruments designed to access emotion has proven problematic (e.g., Heavey et al., 2017). Drawing on discursive psychological research, we suggested that even attempts to refine these instruments to capture mixed emotions have the same limitations, assuming that feelings can be accurately intuited and described retrospectively.

We noted that conversation analysts have highlighted the importance of a range of interactional elements that shape how emotions become meaningful for participants. These intricate details are notoriously difficult for research participants to recall and reconstruct. Further, discursive psychologists have shown how emotion avowals and ascriptions construct and respond to actions such as accounts, complaints, and arguments. Despite these insights, descriptions of emotions on scales or in interviews continue to be overwhelmingly treated as neutral representations of inner experiences.

Emotionography offers an alternative approach to the study of emotion by examining how it unfolds in actions and sequences between different parties, across varying everyday and institutional settings, and different practices within those settings. We illustrated the power and relevance of this approach by addressing the contemporary emotion research topic of mixed emotion, focusing on a notable environment of organized helping. This involves respecifying what we understand by emotion, whether mixed or not. Mixed emotion in traditional work has been identified through self-reports, facial expressions, physiological responses, and qualitative interviews, often combining such methods to triangulate data to access “pristine inner experiences.” Our approach is focused on emotion as displayed in and through interaction because this is the primordial site where it becomes live. In our prior research, we noticed that there are occasions where both crying and laughing occur together. The analysis above was designed to explain what might happen when such mixtures occur.

### Findings from our analysis of laughing with crying in organized helping

Hepburn (2004) tracked the way upset was displayed in naturally occurring telephone conversations, noting that more conventional signs such as sobs were combined with wet sniffs, breathiness, pitch elevation, and tremulous delivery of specific words. Our prior research showed that upset is mostly not produced as a separate action in our helpline calls but is inflected into the delivery of talk in different ways [see Weatherall (2021) for further discussion of talking through upset]. In most of our examples of upset in this helpline, upset occurs when callers deliver accounts of abuse to children and minors, displaying the effect that witnessing the abuse has had on the speaker and interactionally calibrating the severity of the abuse (see Hepburn and Potter, 2007, 2012). However, in our subset of calls where the upset co-occurs with interpolated laughter, there are more complex interactional tasks to be managed, and this is where we see the introduction of laughter particles.

In our examples, laughter was mostly delivered as particles interpolated into keywords or brief particles placed at the completion of specific actions. At times, we analyzed this laughter as highlighting the insufficiency or problem status of certain descriptions, e.g., “starting a new mortgage” in extract 3, with its potential for being heard as more concerned with financial matters than her daughter's welfare. Such descriptions were marked as problematic without, nevertheless, retracting or modifying them (for which there would be a normative set of “repair practices”; e.g., Schegloff et al., 1977). Laughter particles also modulate actions, particularly where they might be heard as disaffiliated or challenging—both extracts 2 and 3 involved resisting advice delivery.

One aspect of our analysis focused on the verbal acknowledgments and self-attributions of emotions, drawing from the field of discursive psychology. In extract 1, we observed Penny's avowal of being upset, highlighting the profound impact her friend‘s house burning had on her. In extract 4, we noted how the caller explicitly avowed feelings of “anger” and “upset,” which conveyed her moral disapproval of her family for withholding important information and positioned her as a victim of this withholding. In both cases, laughter particles managed some of the complexities and potentially problematic aspects of the actions being discussed.

Upset and laughter, then, were delicately placed to navigate different interactional jobs. Therefore, the “mixture” is not paradoxical but a coherent and conversationally focused byproduct of the different practices that make up unfolding action. They are not fighting with one another or canceling each other out. Rather, they are delicately placed to work precisely where they are needed. Indeed, it could be argued that we do not have mixed emotions, but emotional displays and avowals issued at just the right moment.

## Conclusions

Emotionography treats the currency of experience as laid out in language, texts, and embodied actions employed by individuals. In this article, we draw on a tradition inspired by Wittgenstein (1953), Sacks (1992), Schegloff (1992), and Edwards (1997). Emotionography studies emotions as they occur naturally, where their display, reception, attribution, and acknowledgment are public. It is grounded in the perspectives of the interactants displayed in real-time through unfolding talk, working with recorded and transcribed materials that capture the granularity that is essential for the interactants. The specificity of our analysis allows us to issue a challenge to researchers using more conventional methods to provide equally precise accounts that disambiguate such cases.

Some emotion researchers will undoubtedly find this approach unsatisfactory as it is systematically agnostic with respect to “experience” and putative cognitive, physiological, or neuronal aspects of emotion. How is this still studying emotion? The answer is three-fold. First, there is the issue of what is prioritized. If your priority is what is public and consequential about emotion, then emotionography is designed specifically to map this domain. Second, experience, cognitions, and even physiology surface in interaction as participants” issues. Is the caller starting to feel upset? Is the increasing delay in responding a consequence of the upset showing up in constricted vocal cords or blocked mucous membranes? Is the upset about care for an injured child or failure to obtain desired support from social services? The point is that these issues enter emotionography as they become live for participants and are analyzable. Third, as we have noted, the choice is not between directly studying interaction and directly studying experience. In orthodox emotion research, experiences, feelings, or similar things become data when they emerge in descriptions or categorizations generated by the use of more or less structured instruments.

Given that emotion language is pervasively performative, there are considerable challenges in directly addressing experience, as emotion researchers such as Heavey et al. (2017) recognize. It is possible that a better understanding of the public world of emotion practices would help identify ways of improving such measures and highlight areas of problem. For us, however, the public world of emotion is rich, consequential, and, surprisingly, mostly underexplored, and the public world is a central part of the machinery of organized helping.

## Data availability statement

The datasets presented in this article are not readily available because they are confidential recordings of helpline interaction. Requests to access the datasets should be directed to alexa.hepburn@rutgers.edu.

## Ethics statement

All participants gave full consent for calls to be recorded and used for a range of research and teaching purposes, and ethical consent was granted by Nottingham Trent University's Ethics Committee, and followed the helpline's own internal consent procedures. All references to names, places and other identifying features have been anonymized. The studies were conducted in accordance with the local legislation and institutional requirements. Written informed consent for participation was not required from the participants or the participants' legal guardians/next of kin in accordance with the national legislation and institutional requirements.

## Author contributions

All authors listed have made a substantial, direct, and intellectual contribution to the work and approved it for publication.

## Footnotes

^1^Potter, J., and Hepburn, A. (in preparation). *Emotionography: A Method for Analyzing Emotion in Psychology and the Social Sciences*. Washington, DC: American Psychological Association Press.

## Appendix 1

### Selected glossary of emotion-relevant transcription conventions. (adapted from Hepburn and Bolden, 2017)

**Table:**

*Underlining*	emphasis through all of the word: Oh: or part of the word: Linda
*Capitals*	elevated volume: MAYBE
*Degree signs*	reduced volume - preceding the word: °Yeh.
	Or surrounding a string: °here I've godda gid° double degree signs indicate. Whispering or sotto voce*:* °°I hhave°°
*Up arrows*	sharp rises in pitch across a string of words: ↑we pl'se bring↑
*Down arrow*	sharp falls in pitch across a string of words: ↓see yah. Yah.↓
*Underlining*	Slightly elevated pitch (may include volume) on the vowel only: Yes
*Up to down contour*	an underlined vowel followed by a colon: pa:ssing.
*Down to up contour*	an underlined colon: ni:ght
*Creaky delivery*	# before a word: #door enclosing a string of words: #ma best.#~
*Animated delivery*	exclamation marks: ↑G[r:ea ]:t!
**Components of laughter**	
*Voiced vowels*	huh/hah/heh/hih/hoh/ha/ehh/
*Voiced consonants*	‘tsshh' or ‘khuh'
*Plosiveness enclose particles in parenthesis*:	a(h)wa(h)ay / thi(h)nk
*Breathy*	hh-hh-hh or hhhmhhhh or uhhhp
*Smiley voice*	‘£' before a word or enclosing string of words: £cook th‘m£
**Components of upset**	
*Sniffs*	°.snih' (quiet nasal) or ‘.SCHHIH' (loud ‘wet').
*Sobbing*	HHhuhh >.hih .hih <
*Tremulous*	enclose in tildes (~): ~↑I'm ↑sorry.~
*Aspiration*	in words parenthesis (*h*) represents plosive outbreath; *h* represents “breathy” delivery.
Creaky delivery preceded by #:	~THAt wasn' #ma best.#~
